# Gastroesophageal Disease and Environmental Exposure: A Systematic Review

**DOI:** 10.21203/rs.3.rs-4650430/v1

**Published:** 2024-07-31

**Authors:** Daniel Hyun Kim, Sanjiti Podury, Aida Fallah Zadeh, Sophia Kwon, Gabriele Grunig, Mengling Liu, Anna Nolan

**Affiliations:** Department of Medicine, Division of Pulmonary, Critical Care and Sleep Medicine, New York University Grossman School of Medicine; Department of Medicine, Division of Pulmonary, Critical Care and Sleep Medicine, New York University Grossman School of Medicine; Department of Medicine, Division of Pulmonary, Critical Care and Sleep Medicine, New York University Grossman School of Medicine; Department of Medicine, Division of Pulmonary, Critical Care and Sleep Medicine, New York University Grossman School of Medicine; Department of Medicine, Division of Environmental Science, New York University Grossman School of Medicine; Department of Population Health, Division of Biostatistics, New York University Grossman School of Medicine; Department of Medicine, Division of Pulmonary, Critical Care and Sleep Medicine, New York University Grossman School of Medicine

**Keywords:** Systematic Review, Environmental Exposure, Smoking, Particulate Matter, Aerodigestive Disease

## Abstract

Environmental exposure-associated disease is an active area of study, especially in the context of increasing global air pollution and use of inhalants. Our group is dedicated to the study of exposure-related inflammation and downstream health effects. While many studies have focused on the impact of inhalants on respiratory sequelae, there is growing evidence of the involvement of other systems including autoimmune, endocrine, and gastrointestinal.

This systematic review aims to provide a recent update that will underscore the associations between inhalation exposures and upper gastrointestinal disease in the contexts of our evolving environmental exposures. Keywords focused on inhalational exposures and gastrointestinal disease. Primary search identified n = 764 studies, of which n = 64 met eligibility criteria. In particular, there was support for existing evidence that PM increases the risk of upper gastrointestinal diseases. Smoking was also confirmed to be major risk factor. Interestingly, studies in this review have also identified waterpipe use as a significant risk factor for gastroesophageal reflux and gastric cancer.

Our systematic review identified inhalational exposures as risk factors for aerodigestive disease, further supporting the association between environmental exposure and digestive disease. However, due to limitations on our review’s scope, further studies must be done to better understand this interaction.

## INTRODUCTION

Exposome-associated morbidity and mortality is a global health concern. Environmental exposures that individuals encounter over their lifetimes include air pollution, water pollution, diet, and radiation. The exposome’s rate, volume, and variety have been linked to heterogenous negative health effects, but mechanisms remain elusive in many disease states. Studying the exposome provides valuable insights into the interplay between environmental factors and human health.^1^ Of the various environmental exposures, inhalational exposure has been of great interest, especially in the context of rising levels of global air pollution due to global warming, wildfires, wars, and population growth.

Studies investigating the link between environmental exposures and disease have the potential to impact millions globally. Air pollution is associated with 7-million premature deaths annually, and levels have steadily risen over the past few decades.^2–3^ Over half of the world’s population are exposed to levels of air pollution that are substantially above the WHO Air Quality Guidelines.^4^ There are a greater proportion of non-communicable disease attributable to environmental exposure in developing countries that utilize industrial production factories, primary contributors to emissions.^5^

Particulate matter (PM) exposure, in particular, is a global cause of significant aerodigestive morbidity and mortality.^6–46^ The destruction of the World Trade Center (WTC) on September 11, 2001 (9/11) led to intense PM exposure of first responders and inhabitants of New York City (NYC).^15–46^ WTC-PM exposure is associated with obstructive airways disease (OAD). PM was also found to have associations with gastroesophageal diseases, such as gastroesophageal reflux disease (GERD) and Barrett’s Esophagus (BE).^47–49^ Approximately 44% of WTC rescue/recovery workers had developed GERD symptoms by 2005.^50^ In contrast, GERD has a prevalence of 20% in the US.^51 53^ There is also evidence of comorbid GERD and OAD, as WTC-exposed firefighters with OAD had 3-fold higher risk of developing GERD.^49,54^

GERD is the most prevalent gastrointestinal disorder affecting at least 20% of the US population, and leading to substantial morbidity.^55^ Globally, GERD prevalence is 10–25%.^55,56^ Aerodigestive complications also include BE, and malignancy such as esophageal adenocarcinoma (EAC).^15,57,58^

Aerodigestive disease can also induce or worsen respiratory disease such as airway hyperreactivity (AHR). This may be explained by the clearing mechanism of the respiratory system and its close proximity to the digestive system at the pharynx. However, this is an area of active investigation.^59^ Prior systematic reviews on the topic have only focused on single inhalational exposures and/or single diseases/outcomes.^60–65^

Our systematic review focuses on the exposome in a more holistic manner in order to assess its effects on the scope of gastroesophageal diseases on a greater scale. We investigated the associations between exposure (particulate matter, smoking, waterpipe smoking) with diseases of the upper gastrointestinal tract (GERD, Barrett’s and malignancy).

## METHODS

### Review Strategy.

Our systematic review adhered to the Preferred Reporting Items for Systematic reviews and Meta-Analysis (PRISMA) guidelines.^66,67^ Our Population, Exposure, Outcome **(PEO)** question was to investigate among adult populations **(P)** whether there is an association between inhalational exposure (e.g., air pollution, cigarette/tobacco smoke, marijuana smoke, vape/e-cigarette aerosols) **(E)** and esophageal or gastric disorders/disease **(O)**.

PubMed searches were conducted on May 1, 2024 as per the protocol of our systematic review registered on PROSPERO on April 29, 2024 and can be accessed at Prospero ID 536834. The following **MeSH Terms** were searched for using the MeSH Database:

(“Particulate Matter”[Mesh]) OR (“Vaping”[Mesh]) OR (“Smoking”[Mesh]) OR (“Smoking Devices”[Mesh]) OR (“Tobacco Use”[Mesh]) OR (“Tobacco Use Cessation Devices”[Mesh]) OR (“Electronic Nicotine Delivery Systems”[Mesh]) OR (“E-Cigarette Vapor”[Mesh]) OR (“Inhalant Abuse”[Mesh]) OR (“Marijuana Smoking” [Mesh]) OR (“Marijuana Use”[Mesh]) OR (“Marijuana Abuse”[Mesh]) OR (“Cannabis”[Mesh]) OR (“Cannabinoids”[Mesh])

[AND]

(“Stomach Diseases”[Mesh]) OR (“Esophageal Diseases”[Mesh])

The complete listing of associated terms that were searched for with each of the above Mesh terms, can be found at MeSH Database. For example, when searching for “stomach diseases” under its associated Mesh term, we were able to search for Reflux, Gastritis, Peptic Ulcer, Stomach Neoplasms, Zollinger-Ellison syndrome, etc. Reference-list screening was also used.

For this review, we have defined environmental exposure to include PM_2.5_, PM_10_, tobacco/cigarette smoke, vape/e-cigarette aerosols, and marijuana/cannabinoid inhalation. We have defined esophageal and gastric disease to include the following: gastroesophageal reflux disease (GERD), Barrett’s Esophagus (BE), peptic ulcer disease (PUD), esophagitis, gastritis, esophageal cancer, and stomach cancer.

### Study Criteria.

Studies were included if they **(1)** discussed the environmental/occupational exposure to inhalants, specifically, PM_2.5_, PM_10_, tobacco/cigarette smoke, marijuana smoke, and/or vape/e-cigarette vapor, **(2)** evaluated effects of exposures on esophageal or gastric diseases, **(3)** performed on adult human subjects, **(4)** were written in English, and **(5)** were published within the last 10 years.

Studies were excluded if they **(1)** were not original research, **(2)** consisted of translational research, **(3)** were case reports or series, or **(4)** were conducted on a pediatric population.

### Data Extraction.

Articles were reviewed and data regarding study design, patient characteristics, sample size, and tool used were extracted. Results from database searches were filtered for full-text articles, human subjects, English language, and publication date and imported into Endnote X9. Original research papers were reviewed for (title, abstract, and full text) to ascertain eligibility. We examined references cited in the relevant articles. All results were screened by DHK and SP and further independently evaluated by AN. Disagreements were resolved by consensus.

### Risk of Bias Assessment.

Systematic review inherent bias (selection, detection, performance, and reporting) was addressed through study design/search algorithm. Selection bias was addressed by having predetermined inclusion, exclusion criteria and distinct definitions. Detection and performance bias were addressed by having at least two rounds of screening individually performed by DHK and SP. Reporting bias was minimized by using PubMed search filters for peer-reviewed published articles of human subjects written in English and removing duplicates.

The Newcastle-Ottawa Scale (NOS), a domain-based approach was used to assess the degree of bias.^68^ Scales adapted for case-control and cross-sectional studies were used. Total scores obtained by the scale were converted to AHRQ standards or as done in previous studies to reflect the quality of each paper: “good” (green), “fair” or “satisfactory” (yellow), and “poor” or “unsatisfactory” (red).^69^

### Ethics Approval.

This study does not require ethical approval as it involves a review of publicly available research and utilized anonymized original data.

## RESULTS

### Literature Search.

**Figure 1:**
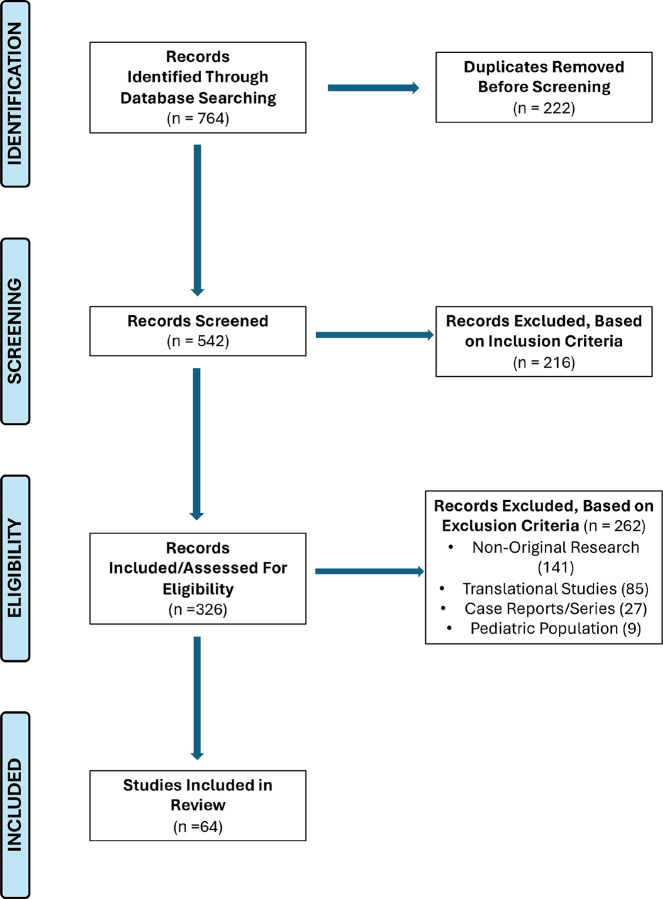
Study design per Preferred Reporting Items for Systematic Reviews and Meta-Analyses (PRISMA) Guidelines. PRISMA is an evidence-based minimum set of items for reporting in systematic reviews and meta-analyses.^66,67^

A total of 764 studies were identified from PubMed and reference-list screening, Fig. 1. After removal of 222 duplicates, 542 articles were screened. Following application of inclusion criteria, 216 articles were excluded and 326 articles were assessed for eligibility based on exclusion criteria. Application of exclusion criteria involved removal of 141 non-original research articles, 85 translational studies, 27 case reports/series, and 9 pediatric studies for a total of 262 articles. N = 64 original research articles were considered eligible and included in this review. Data from screening and extraction are available, **Supplemental Tables 1–6**.

Risk of bias using NOS was assessed in cohort (N = 20), case-control (N = 23), and cross-sectional studies (N = 18), **Supplemental Table 7**. Two studies that were case-crossover studies and one study that was an ecological study were unable to be assessed for risk of bias as the NOS and our adaptations did not cover for these types of studies. Scores obtained from the NOS were adapted as in previously published studies to reflect the quality of each paper.^69^ Cutoffs for each risk of bias assessment depending on article type can be found within the footnote of **Supplemental Table 7**. Among cohort studies, N = 15 articles were of good quality, N = 1 of fair quality, and N = 4 of poor quality. Among case-control studies, N = 15 were of good quality, N = 5 of fair quality, and N = 3 of poor quality. Among cross-sectional studies, N = 14 were of good quality, N = 2 of satisfactory quality, and N = 2 of unsatisfactory quality.

### Study Characteristics.

**Figure 2:**
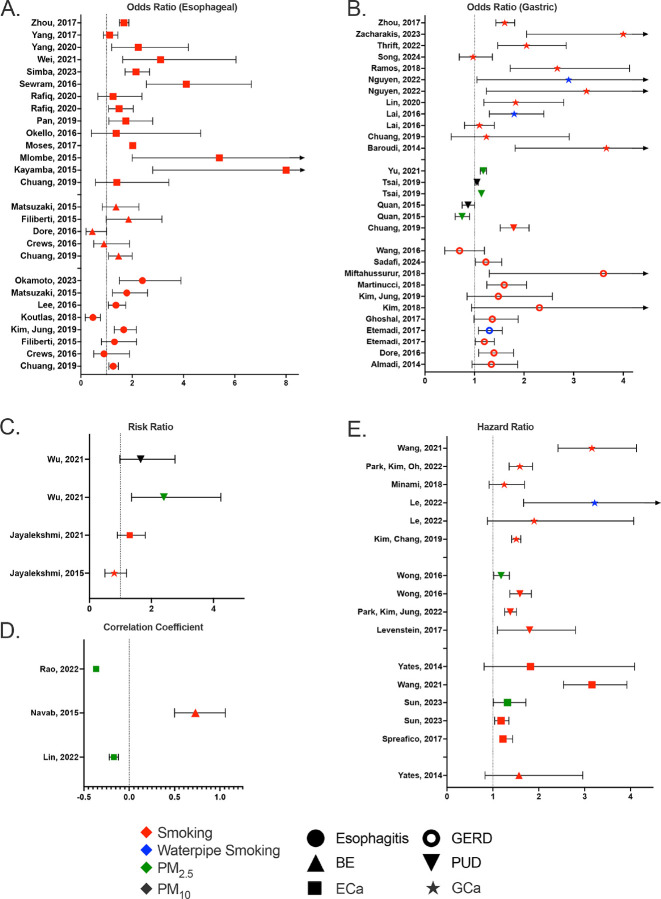
Overview of Data Synthesis: (**A**) Summary of odds ratios for esophageal diseases (esophagitis, BE, ECa), (**B**) Summary of odds ratios for gastric diseases (GERD, PUD, GCa), (**C**) Summary of risk ratios, (**D**) Summary of correlation coefficients, (**E**) Summary of hazard ratios.

The populations of patients with esophageal or gastric disease included those afflicted with esophagitis (n = 8), BE (n = 8), ECa (n = 24), GERD (n = 11), PUD Fig. 1. Study design per Preferred Reporting Items for Systematic Reviews and Meta-Analyses (PRISMA) Guidelines. PRISMA is an evidence-based minimum set of items for reporting in systematic reviews and meta-analyses.^66,67^

(n = 9), and GCa (n = 16). Studies that focused on any other outcomes did not meet exclusion/inclusion criteria. The exposures that were investigated in particular were smoking, waterpipe smoking, and PM_2.5_/PM_10_ exposure. While there were no studies that focused on marijuana smoking or vaping/e-cigarettes that met our inclusion/exclusion criteria we know from the literature that use of cannabinoids and vaping are linked to the development of gastrointestinal disorders.^134^ One study investigated the role of exposure to second-hand smoke, in addition to direct cigarette smoke exposure.^110^ Most studies produced an odds ratio (OR), risk ratio (RR), correlation coefficient (CC), or hazard ratio (HR) to measure each of the risks associated with their respective exposures for a particular outcome, of which are summarized in Fig. 2 (see raw data in **Supplemental Table 8A-E**). Among those studies, some reported using adjusted models in their analyses. Additionally, other studies focused on the percent presentation of risk factors;^72,86^ risk by measuring the increase in incidence of the respective disease;^91–93,114^ the differences in mortality with respect to magnitude of exposure^95^, and utilized a novel predictive model to identify risk factors.^115^ Table 1.

### Esophagitis.

Current tobacco use was identified as a significant risk factor for reflux esophagitis (RE).^73,77,97,104^ When studying gender-specific differences between smoking and risk of RE, Kim, Jung, et al found that smoking led to greater risks of RE among women compared to men.^84^ Associations between smoking and eosinophilic esophagitis (EE) were also investigated. Some studies found that EE were significantly less likely to have ever smoked cigarettes compared to non-EE controls, but smoking was not significantly associated with increased risk of EE.^74,86^ Lee, et al, however, identified smoking as a significant risk factor for asymptomatic EE (AEE).^89^

### Barrett’s Esophagus.

Smoking was identified as a risk factor for BE.^73,97^ Schmidt, et al found that BE cases were significantly more likely to smoke.^114^ Navab, et al found positive correlation between current and prior tobacco use and BE.^102^ Etemadi, et al found associations between smoking and BE that were independent of intensity, age at initiation, and GERD, but dependent on duration and years since cessation.^76^ Other studies, however, produced conflicting results: some studies found that current and former smoking were not significantly associated with BE.^75,130^

### Gastroesophageal Reflux Disease.

Multiple studies identified smoking as a risk factor for GERD.^75,78,96,98,113^ Kim, Jang, et al interestingly found that former smoking was significantly associated with risk of GERD, while current smoking was not significantly associated.^83^ When investigating gender-specific differences on the effects of smoking on the risk of GERD, Kim, Jung, et al found that smoking increased risks in both men and women.^84^

One study investigated the effects of waterpipe smoking in addition to traditional cigarette smoking on the risk of GERD. Etemadi, et al found that waterpipe smoking was most strongly associated with “severe and frequent” reflux, and prevalence of the disease was associated with waterpipe use and duration. In addition, they found that cigarette smoking was a significant risk factor of any form of reflux among men.^76^

Conversely, some studies noted no significant association between GERD and smoking. Almadi, et al observed a higher prevalence of GERD among smokers than non-smokers, but found no significant difference.^70^ Wang, Leena, et al also did not find any association between cigarette smoking and risk of GERD.^123^

One study, Seo, et al, developed a prediction model that was significantly able to predict GERD-related medical utilization in the South Korean population and identified PM_2.5_ as a risk factor for GERD.^115^

### Peptic Ulcer Disease.

Chuang, et al identified current tobacco use as a significant risk factor for PUD and that higher cumulative amounts of tobacco use were at higher risk for PUD.^73^ Further, Begovic, et al found that more than half of ulcer patients enrolled into their study were smokers, and this difference was significant when compared to the those who were non-smokers.^72^ Levenstein, et al observed that age-, gender-, and socioeconomic status-adjusted associations were significant for smoking.^90^

Park, Kim, Jung, et al investigated the role of changes in smoking status in risk of gastroduodenal ulcer. They observed that changes in smoking status from never smoker, quitter, and current smoker, to current smoker in particular had relatively higher HRs than other groups. When comparing smoking amount levels, they found that smokers who smoked > 20 pack-years had significantly higher risk than lighter smokers.^107^

Other studies had investigated the role of PM exposure in risk of PUD. Tsai, et al found that increases in both PM_2.5_ and PM_10_ were significantly associated with increased risk of PUD hospitalizations on warm days, but only PM_10_ was significantly associated on cold days.^122^ Similarly, Wong, et al found that PUD hospitalization was associated with 10 ug/m^3^ increases in PM_2.5_. When investigating different types of ulcers, they found that associations with PM_2.5_ were significant for gastric ulcers, but not for duodenal ulcers.^126^ Wu, et al observed that cumulative RRs for PM_2.5_ and PM_10_ showed nearly linear adverse effects.^127^ When looking at gender-adjusted differences, significant associations for men and women were only observed for PM_2.5_.

Quan, et al found that when air pollution exposures were assessed over 3-, 5-, and 7-day averages, pollutants were inversely associated with upper gastrointestinal bleeding (UGIB).^109^ Yu, et al observed a potential dose-response relationship between quartile concentrations of PM_2.5_ one month prior to detection of PUD. Subjects in the highest quartile of PM_2.5_ exposure displayed significantly higher risk and the detection of PUD was associated with a 10 ug/m^3^ in PM_2.5_.^131^

### Esophageal Cancer.

Many studies found associations between smoking and ECa.^101,116,133^ Other studies focused on esophageal squamous cell carcinoma and also identified smoking as a risk factor and this risk increased with tobacco intensity and smoking duration, but no significant difference with respect to macroscopic type of cancer, as smoking showed similarly increased risks for both ulcerative type and medullary type eosinophilic squamous cell carcinoma (ESCC).^81,85,100,117,125,128^ Jayalekshmi, et al observed higher risks of ESCC for current bidi and cigarette smokers.^80^ Conversely, some studies observed non-significant relationship between inhalational exposures and ECa.^73,105,129^

Some studies looked at how smoking affected survival for those afflicted with ECa. Spreafico, et al found that smoking conferred worse overall survival in the combined Boston-Toronto Cohort for each 20 pack-year increase.^119^ Other observed how current and former smoking contributed to decreased survival with respect to subtype, specifically ESCC and esophageal adenocarcinoma (EADC).^124,130^

One study in particular, Rafiq, et al evaluated both smoking and second-hand smoke as a risk factor for ECa, with increased risks associated with either exposure.^110^ Another study, Pan, et al focused on associations between smoking and esophageal precancerous lesions (EPL) and found that consuming more than 30 cigarettes/day or having 40 or more pack-years of cumulative smoking was significantly associated with EPL.^106^

Other studies investigated the relationship between PM_2.5_ exposure and ECa. Li, Guo, et al observed a significantly positive association between PM_2.5_ and ECa incidence. When investigating the corresponding lag effects on ECa incidence, they found that a lag effect of 4 years showed the greatest risk for males and females.^91^ Li, Jing, et al examined the modifying effects of urbanization and socioeconomic factors and found a stronger association between PM_2.5_ and incidence for low urbanization groups, and this association was stronger for females than males.^92^ Li, He, et al identified long-term exposure to black carbon, organic carbon, nitrate, and ammonium to be significantly associated with ECa.^93^ Rao, et al found that although spatial distributions of hospitalization rate of ECa in 2016 were not consistent with that of PM_2.5_ concentration in the same year, concentrations of PM_2.5_ in 2003 and 2004 had the strongest correlations with hospitalization rate in 2016.^112^ Sun, et al observed a linear concentration-response relationship between long-term PM_2.5_ and ECa.^120^ Conversely, Lin, Shih, et al observed that the average number of deaths due to ECa decreases with increasing average PM_2.5_ concentration.^95^

### Gastric Cancer.

As with the previous outcomes, most studies identified smoking as a risk factor for GCa.^71,111,132,133^ Current cigarette smoking status was found to be attributed to increased risk of GCa and this risk increased among those with longer durations of smoking or later starting ages of smoking.^94^ Current smoking was also found to have increased risk of stomach cancer death.^99^ When assessing changes in smoking status, one study found that those who changed to their current status to “smoking” showed increased risk of GCa and this risk was the highest in heavier smokers.^108^ One study found that smoking was only significantly associated with single GCa and synchronous multiple gastric cancer (SGMCa) in advanced gastric cancer (AGCa) patients.^118^ Current smoking also showed increased risk for gastric adenocarcinoma (GAC) and gastric non-cardia adenocarcinoma (GNCA).^124^ Interestingly, Jayalekshmi, et al found that bidi smoking was significantly associated with GCa risk, but cigarette smoking was not. This risk increased with the number of bidis smoked daily and with duration of bidi smoking.^79^ Conversely, Chuang, et al found that tobacco use was a non-significant risk factor for GCa.^73^ Other studies found that current smoking increased risks of intestinal metaplasia for both men and women. Further, this risk increased with increasing duration and total dose.^82,121^

Some studies investigated the role of waterpipe smoking in GCa risk. Several studies in Vietnam showed that water pipe smoking was positively associated with GCa risk, but there was no significant interaction between the effects of water pipes and cigarette smoking on GCa risk.^87,88,103^ Li, He, et al found that long-term exposure to black carbon, organic carbon, nitrate, ammonium, and sulfate was significantly associated with stomach cancer.^93^

## DISCUSSION

In this systematic review, we investigated the associations between environmental exposures and diseases of the upper gastrointestinal tract. Through a comprehensive review of the available literature, we identified complex relationships between environmental exposures and upper gastrointestinal diseases. Most of the studies showed that exposures including particulate matter, smoking, and waterpipe were significantly associated with higher risk of aerodigestive diseases.

Particulate matter (PM) exposure is a global cause of significant pulmonary morbidity and mortality.^6–46^ Our review supports existing evidence suggesting that exposure to PM may also increase the risk of diseases affecting the upper gastrointestinal tract. Studies included in this review demonstrated links between PM exposure and an increased risk of ECa and PUD, although the underlying mechanisms remain to be fully explained. These findings highlight the importance of considering environmental factors, such as air pollution, in the context of upper gastrointestinal health. PM consists of various harmful compounds which can trigger inflammatory responses, oxidative stress and DNA damage that contribute to the development of cancer and ulceration. Moreover, studies showed that PM may disrupt the gut microbiota, leading to increased risk of gastrointestinal inflammation and cancers.^135^

Cigarette smoking has been recognized as a major risk factor for various cancers, including those of the gastrointestinal tract. Consistent with previous research, our review highlights the detrimental effects of smoking on the upper gastrointestinal tract, with a notable association observed between smoking and an elevated risk of Barrett’s esophagus, GCa, ECa and PUD. The carcinogenic effect of smoking is attributable to mutations in critical genes caused by tobacco metabolites and chemicals. Smoking is also associated with progression, aggressiveness, and reduced survival rates of existing gastrointestinal cancers. Smoking may be associated with exacerbation of GERD symptoms due to reducing esophageal sphincter tone and increasing gastric acid production.^136^

Waterpipe smoking has increased worldwide due to a perception that it is less harmful than cigarette smoking. However, waterpipe smoke contains tobacco and several toxicants that may increase the risk of developing aerodigestive disease, as identified in our review. Numerous carcinogens have been identified in waterpipe smoke including polycyclic aromatic hydrocarbons (PAHs), volatile aldehydes, and heavy metals, which can cause DNA damage and develop cancer over time.^137^ Moreover, emerging evidence suggests that vaping and marijuana use may also impact gastrointestinal health, although further investigation is warranted to better understand the nature of these associations.

Gastrointestinal symptoms associated with vaping can occur in more than half of exposed patients.^138,139^ The gastrointestinal symptoms are thought to be directly related to the inhalation of nicotine; users of novel nicotine delivery products (vapers) usually take in higher doses of nicotine than tobacco smokers.^140^ Nausea, vomiting, diarrhea and abdominal pain are also signs of with E-cigarette or Vaping Product Use-Associated Lung Injury (EVALI), as indicated in several case reports.^141^ In a survey of UK vapers (that met our exclusion criteria), the incidence of the new symptom vomiting amongst “current vapers” was 13.0% and 21% for nausea. Current vapers using cannabinoid-based substances reported nausea significantly more frequently than other groups. Respondents reporting vomiting/nausea were given the diagnosis of gastritis or gastroenteritis. The incidence of nausea and vomiting was not increased in exclusive vapers compared to concurrent smokers and vapers.^142^

In tobacco smokers, due to the burning process, nicotine can be transformed into nitrosamines via nitrosation, and many of these nitrosamines, such as nicotine-derived nitrosamine ketone (NNK) and N-nitrosonornicotine (NNN), are potent carcinogens linked to esophageal and stomach cancer.^143,144^ Novel delivery devices such as e-cigarettes produce 5% as much nitrosamines compared to standard burning tobacco products, leading to an assumption that e-cigarettes are safer than cigarettes and could be used as cessation aids. However, translational *in vitro* and murine studies showed that nicotine from e-cigarette induced carcinogenic DNA-adducts and inhibited DNA repair just like nicotine-derived nitrosamine ketone (NNK). Because it often takes over two decades for tobacco smokers to develop cancer, mice were exposed to e-cigarette vapors for one year and had their organs examined. While cancers were detected in the lungs of the mice due to e-cigarette exposure, cell hyperplasia also occurred in the bladder epithelium, raising the possibility that although e-cigarette exposure is inhalational, it can cause systemic cancers.

Furthermore, the nicotinic acetylcholine receptor (nAChR), a genetic variant of which is consistently linked to lung cancer in large genetic studies, might mediate carcinogenesis through directly binding nicotine (and nitrosamines) in airway epithelium. This mechanism could provide direct carcinogenesis of nicotine and nicotine-metabolites to all cells that express the nAChR, particularly in carriers of the variants that are associated with tobacco smoking and cancer.^145–148^ Following the idea that inhaled nicotine could produce carcinogenic molecules in human users, an untargeted metabolomics analysis of urine demonstrated a trend of increased carcinogen biomarkers in the samples of a relatively small cohort of vapers (n = 34 vs. n = 45 non-users).^149^

### Limitations

While this systematic review provides valuable insights into the associations between environmental exposures and upper gastrointestinal diseases, the included studies vary in design, methodology, and population characteristics, which may introduce heterogeneity and bias. Some studies used adjusted models when calculating ORs or HRs (aOR; aHR). It is possible that these adjustments are complex and vary widely across these studies, further contributing to heterogeneity. Additionally, the majority of studies are observational in nature limiting causal inference and necessitating further research, including prospective cohort studies and mechanistic investigations. This study only relied on the PubMed database for the identification of potentially eligible studies. Our risk of bias assessment (NOS) was able to evaluate the majority but not all studies assessed in this review.

Other limitations revolved around how we defined environmental exposure and aerodigestive disease as a whole. Our study defined environmental exposures as air pollution in the form of particulate matter, cigarette/tobacco smoke, marijuana smoke, vape/e-cigarette aerosols. Due to this, it was not possible to completely cover the entire scope of environmental exposures that afflict society. In addition, our definition of aerodigestive disease focused on diseases of the upper gastrointestinal tract, which comprised of esophagitis, Barrett’s esophagus, GERD, PUD, and esophageal/gastric cancer based on the articles we found. It is very likely that there are other aerodigestive diseases that interact strongly with environmental exposures that were not covered by this paper. Due to these definitions and our inclusion/exclusion criteria, we also found no eligible articles that investigated the interactions of marijuana smoke and vape/e-cigarette aerosols with aerodigestive disease.

### Future Research

Currently, there are no human studies available to clearly define the cancer-inducing potential of non-burning nicotine delivery products such as e-cigarettes. However, this could be due to the very extensive lag time between carcinogen exposure and clinical cancer diagnosis in humans. Future studies could expand our definitions to account for interactions not present within this review, such as those of marijuana smoking, vaping, and those of the lower intestinal tract. Additionally, future studies could assess the contribution of specific occupational exposures to aerodigestive health, as this study only focused on exposures commonly experienced by the general population. Such additional exposures include asbestos, synthetic fiber dust, chrysotile dust, nephrite, and potentially harmful elements (PHEs) which are all commonly present in mining or textile industries and in developing societies in general. Such investigations could yield valuable insights for those whose occupation or geographic location puts them at risk for such diseases, as aerodigestive disease is often not recognized for those working/living under such conditions. In addition, this could identify how specific exposures incite disease in various cohorts.

### Conclusion

The implications of these findings are significant from both a public health and clinical perspective. Efforts to reduce exposure to environmental pollutants, such as particular matter, could potentially mitigate the burden of upper gastrointestinal diseases in affected populations. Similarly, targeted interventions aimed at reducing smoking behavior and promoting smoking cessation may help reduce the incidence of BE and malignancy. Furthermore, continued research into the potential health effects of emerging trends, such as vaping and marijuana use, is crucial for informing preventive strategies and improving patient outcomes.

This review provides support for the connection between environmental exposures and digestive health, which is especially important considering that those who have been exposed to environmental/occupational inhalants are generally not covered for their digestive health. We hope that this review will promote further recognition of treatment of digestive disease with inhalational exposure.

In conclusion, this systematic review contributes to our understanding of the interplay between exposure to inhalational exposures and diseases of the upper gastrointestinal tract. By synthesizing existing evidence and identifying knowledge gaps, this study highlights the need for approaches to address environmental risk factors and promote gastrointestinal health.

## Figures and Tables

**Table 1:** Study Characteristics

Study		Country	Exposure/Design	Study Size/Time Period	Outcome of Interest	Relevant Findings
1	**Almadi, 2014** ^70^	Saudi Arabia	Smoking Cohort Study	4 shopping centers, Riyadh / N = 1,265 Dec. 2012–Jan. 2013	**GERD**	Higher prevalence of GERD in smokers (51.63% vs. 44.41%), but not significant (p = 0.09) No significant association between GERD and smoking (OR: 1.34; 95% CI: 0.95–1.87)
2	**Baroudi, 2014** ^71^	Tunisia	Smoking Case-Control Study	Salah Azaiez Insititue of Oncology N = 348 2009–2010	**GCa**	Smoking more than 10 cigarettes a day is significantly associated with an increased risk in gastric cancer (OR: 3.66; 95% CI: 1.82–7.78).
3	**Begovic, 2015** ^72^	North Macedonia	Smoking Cohort Study	2 University Clinics / N = 67 2014	**PUD**	Smoking is an important risk factor and more than half of ulcer patients were smokers (65.75%). Percent difference in relation to those who are non-smokers is statistically significant (p = 0.0000).
4	**Chuang, 2019** ^73^	Taiwan	Smoking Cross-Sectional Study	4 hospitals in SW Taiwan / N = 8,135 Apr. 2008–Dec. 2013	**E / BE / ECa / PUD / GCa**	Current tobacco use was a significant risk factor for RE (aOR: 1.26; 95% CI: 1.09–1.46), BE (aOR: 1.47; 95% CI: 1.08–2.00) and PUD (1.79; 95% CI: 1.52–2.10), but nonsignificant for ESCC (aOR: 1.40; 95% CI: 0.57–3.43) and GC (aOR: 1.24; 95% CI: 0.53–2.91). Higher cumulative amounts of tobacco use were at higher risk for PUD (aOR: 1.92; 95% CI: 1.60–2.31)
5	**Crews, 2016** ^74^	US	Smoking Cohort Study	Olmsted County, MN N = 205 Apr. 2011–Oct. 2013	**E / BE**	In a univariate analysis, ever tobacco use was not a significant risk factor for EE/BE (OR: 0.9; 95% CI: 0.5–1.9)
6	**Dore, 2016** ^75^	Italy	Smoking Cohort Study	Sassari, Sardinia / N = 5156 Jan. 2002–Dec. 2013	**BE / GERD**	Adjusted ORs of BE and GERD for current smokers were 0.447 (95% CI; 0.199–1.002) and 1.392 (95% CI: 1.085–1.787), respectively.
7	**Etemadi, 2017** ^76^	Iran	Smoking / Waterpipe Smoking Cohort Study	Valashahr, Fars / N = 9264 2012–2017	**GERD**	Strongest associations of waterpipe smoking were with ‘severe and frequent reflux’ (OR: 1.30; 95% CI: 1.08–1.56) Former use had a stronger association with ‘severe reflux’ and (OR: 1.29; 95% CI: 1.06–1.56) and current use with ‘frequent reflux’ (OR: 1.18; 95% CI: 1.03–1.36). Current cigarette use was a significant risk factor for”any reflux” among men (OR: 1.20; 95% CI: 1.02–1.40) Increases in reflux prevalence associated with waterpipe use duration and intensity.
8	**Filiberti, 2015** ^77^	Italy	Smoking Case-Control Study	12 endoscopic units N = 1420 Mar. 2009–Oct. 2012	**E / BE**	Associations shown between smoking and BE that was independent of intensity, age at initiation, GERD and dependent of duration and years since cessation Among current smokers who smoke >18 cigarettes/day, ORs for RE and BE were 1.31 (95% CI: 0.80–2.17) and 1.86 (95% CI: 0.98–3.16), respectively. Risk of BE significantly increased for those who had smoked for > 32 years (OR: 2.44; 95% CI; 1.33–4.45) and those whom < 9 years have passed since quitting (OR: 2.11; 95% CI: 1.19–3.72)
9	**Ghoshal, 2017** ^78^	India	Smoking Cross-Sectional Study	Uttar Pradesh, Jaunpur District / N = 2876	**GERD**	On univariate analysis, tobacco smoking (105 35.2%] vs. 672 [27.1%]) was associated with GERD. On multivariate analysis, tobacco smoking (OR: 1.36; 95% CI: 0.99–1.88) was associated with GERD
10	**Jayalekshmi, 2015** ^79^	India	Smoking Cohort Study	Karunagappally Cohort, Kerala / N = 65553 men 1990–2009	**GCa**	Bidi smoking was significantly associated with GCa risk (RR: 1.6; 95% CI: 1.0–2.5; P = 0.042), but cigarette smoking was not (RR: 0.8; 95% CI: 0.5–1.2) Bidi smoking increased risk of GCa among never cigarette smokers (RR: 2.2, 95% CI: 1.3–4.0) GCa risk increased with the number of bidis smoked daily (P = 0.012) and with duration of bidi smoking (P = 0.036)
11	**Jayalekshmi, 2021** ^80^	India	Smoking Cohort Study	Karunagappally Cohort, Kerala / N = 65528 men Jan. 1990–Dec. 2013	**ECa**	RRs for current bidi and cigarette smokers were 1.4 (95% CI: 0.98–2.12) and 1.3 (95% CI: 0.9–1.8), respectively. Higher risks for ESCC observed for current bidi smokers (RR: 2.2; 95% CI: 1.3–3.8) and cigarette smokers (RR: 1.6; 95% CI: 1.0–2.5)
12	**Kayamba, 2015** ^81^	Zambia	Smoking Case-Control Study	University Teaching Hospital, Lusaka / N = 100 Oct. 2013–May 2014	**ECa (ESCC)**	Ever smokers showed greater risk of developing ESCC (OR: 8.0; 95% CI: 2.822.7) Much greater proportion of cases than controls (38% vs 0%) were current smokers (p < 0.000)
13	**Kim, Chang, 2019** ^82^	South Korea	Smoking Cohort Study	Kangbuk Samsung Hospital, Seoul / N = 199235 Jan. 2011–Dec. 2017	**GCa (IM)**	For current smokers, the multivariable-adjusted HR for men and women were 1.51 (95% CI: 1.41–1.61) and 0.94 (0.73–1.22), respectively.
14	**Kim, Jang, 2018** ^83^	South Korea	Smoking Cohort Study	N = 2368 Mar. 2013–Dec. 2015	**GERD**	Former smokers showed a significantly greater risk of GERD (OR: 1.93; 95% CI: 1.12–3.35). Current smokers showed a nonsignificantly greater risk of GERD (OR: 2.31; 95% CI: 0.94–5.66).
15	**Kim, Jung, 2019** ^84^	South Korea	Smoking Cross-Sectional Study	Ewha Womans University Mokdong Hospital / N = 10158 Jan. 2015–Dec. 2016	**E / GERD**	Among men, smokers yielded ORs for RE and GERD of 1.67 (95% CI: 1.30–2.16) and 1.48 (95% CI: 0.85–2.57), respectively. Among women, smokers yielded ORs for RE and GERD of 3.47 (95% CI: 1.61–7.48) and 1.35 (95% CI: 0.68–2.67), respectively.
16	**Koca, 2015** ^85^	Turkey	Smoking Case-Control Study	Erzurum Regional Training and Research Hospital, Anatolia / N = 408 Jan. 2008–Mar. 2014	**ECa**	Smoking (X^2^ = 7.629; p = 0.022) was significantly higher in the patient group than the control group.
17	**Koutlas, 2018** ^86^	US	Smoking Case-Control Study	University of North Carolina / N = 340 2011–2015	**E**	EE cases were less likely to have ever smoked cigarettes compared to endoscopy-based non-EE controls (23% vs. 47%, P < 0.001). aOR for ever-smoking was 0.47 (95% CI: 0.17–0.76).
18	**Lai, 2016** ^87^	Vietnam	Smoking / Waterpipe Smoking Case-Control Study	3 major hospitals, Hanoi / N = 1082 Feb. 2003–Apr. 2011	**GCa**	WPT smoking was positively associated with GCa risk. Significantly high GCa risk in current WPT smokers (OR: 1.8; 95% CI: 1.3–2.4) Current cigarette smoking was not a significant risk factor for GCa (OR: 1.1; 95% CI: 0.8–1.4) No significant interaction between effects of WPT and cigarette smoking on GCa risk.
19	**Le, 2022** ^88^	Vietnam	Smoking / Waterpipe Smoking Cohort Study	3 Northern Vietnam Provinces / N = 25619 2008–2019	**GCa**	Significantly higher GCa mortality among ever-smokers than never-smokers (aHR: 2.43; 95% CI: 1.35–4.36) Exclusive WPT smokers showed the highest risk (HR: 3.22; 95% CI: 1.67–6.21), followed by smokers of both WPT and cigarette (HR: 1.99; 95% CI: 0.89–4.63), then exclusive cigarette smokers (HR: 1.90; 95% CI: 0.88–4.07).
20	**Lee, 2016** ^89^	South Korea	Smoking Case-Control Study	Konkuk University Medical Center / N = 2961 Jan. 2010–Jun. 2014	**E (Asymptomatic)**	Current smoking was an independent predisposing factor for AEE (OR 1.366; 95% CI: 1.068–1.748)
21	**Levenstein, 2017** ^90^	Denmark	Smoking Cohort Study	Copenhagen County / N = 3365 1982	**PUD**	Age-, gender-, and socioeconomic status-adjusted associations were significant for smoking (HR: 1.8; 95% CI: 1.1–2.8).
22	**Li, Guo, 2022** ^91^	China	PM_2.5_ Cross-Sectional Population Study	388 cancer registry institutes, Mainland China 2007–2015	**ECa**	Significantly positive association between PM_2.5_ and EC incidence. Lag effect of 4 years showed the greatest risk for males and females at 1.32% (95% CI: 1.20–1.45%) and 2.70% (95% CI: 2.49–2.92%), respectively.
23	**Li, Jing, 2021** ^92^	China	PM_2.5_ Cross-Sectional Population Study	213 Prefectural Level Cities, Mainland China 2000–2015	**ECa**	Stronger association between PM_2.5_ and incidence observed in low urbanization groups, and association was stronger for females than males.
24	**Li, He, 2024** ^93^	China	PM_2.5_ Cross-Sectional Study	Jiangsu Province / N = 524019 2015–2020	**ECa/GCa**	Long-term exposure to black carbon, organic carbon, nitrate, and ammonium was significantly associated with esophageal and stomach cancer. Sulfate exposure was significantly associated with stomach cancer.
25	**Lin, Wu, 2020** ^94^	China	Smoking Case-Control Study	Xianyou County, Fujian Province / N = 1244 Mar. 2013–Jan. 2017	**GCa**	Current cigarette smoking status was attributed to 83% increased risk of GCa (OR: 1.83, 95% CI: 1.19–2.80) Smokers with longer duration of smoking (≥ 20 years) or started at later age (≥ 20 years) had nearly twofold increased risk of GCa vs. nonsmoker (OR: 1.97; 95% CI: 1.28–3.04, OR: 2.02; 95% CI: 1.30–3.14, respectively).
26	Lin, Shih, 2022* ^95^	Taiwan	PM_2.5_ Cross-Sectional Population Study	Entire Population of Taiwan / N = 23.57 million 2010–2017	**ECa**	Average number of deaths from esophagus cancer decreases 0.17 (95% CI: −0.22, −0.12) per 100,000 people with increasing average PM_2.5_ concentration.
27	**Martinucci, 2018** ^96^	Italy	Smoking Cross-Sectional Study	University of Pisa / N = 3012 Oct. 2016–May 2017	**GERD**	In the set of students with GERD, percentage of smokers was higher. In a multivariate analysis, smoking status showed an increased risk of GERD (OR: 1.6; 95% CI: 1.25–2.05)
28	**Matsuzaki, 2015** ^97^	Japan	Smoking Case-Control Study	Keio University Hospital / N = 2608 Oct. 2012–Nov. 2013	**E / BE**	Current smoking showed risks for RE and BE of OR: 1.79 (95% CI: 1.23−2.60) and OR:1.37 (0.83−2.26), respectively.
29	**Miftahussurur, 2018** ^98^	Indonesia	Smoking Case-Control Study	Surabaya / N = 104 Oct. 2014–Nov. 2015	**GERD**	Smokers had a significantly higher risk of GERD compared to non-smokers (OR: 3.60; 95% CI: 1.298–9.955)
30	**Minami, 2018** ^99^	Japan	Smoking Cohort Study	Miyagi Cancer Center Hospital / N = 1576 Jan. 1997–Dec. 2010	**GCa**	Current smokers had increased risk of stomach cancer death in a multivariate adjusted model (HR: 1.25; 95% CI: 0.92–1.69).
31	**Mlombe, 2015** ^100^	Malawi	Smoking Case-Control Study	2 tertiary teaching hospitals / N = 276 Jan. 2011–Feb. 2013	**ECa**	In unadjusted analysis, odds of developing ESCC was 11.2 times higher among smokers than non-smokers, and in adjusted analysis it was 5.4 times higher. OR: 11.2 (95% CI: 5.2–24.0) and aOR: 5.4 (2.0–15.2)
32	**Moses, 2017** ^101^	Malawi	Smoking Cohort Study	Kamuzu Central Hospital, Lilongwe / N = 172 Jun. 2009–Sept. 2012	**ECa**	Esophageal cancer was among the commonest cancers in the cohort (n = 172; 34.5%). Patients with esophageal cancer were more likely to be smokers (OR: 2.02).
33	**Navab, 2015** ^102^	US	Smoking Cross-Sectional Study	Tertiary care center, PA N = 158 1999–2008	**BE**	Correlation coefficients for current and prior tobacco use were 0.73 (95% CI: 0.50–1.06) and 0.92 (0.64–1.31), respectively.
34	**Nguyen, 2022** ^103^	Vietnam	Smoking / Waterpipe Smoking Case-Control Study	Bach Mai Hospital / N = 226 Jan. 2018–Dec. 2018	**GCa**	Compared to never tobacco smokers, risk of GCa significantly increased among tobacco smokers (OR: 2.95; 95% CI: 1.26–6.90, p = 0.013) For types of tobacco, increased risk was observed in exclusively cigarette smokers (OR: 3.26; 95% CI: 1.24–8.55, p = 0.017) and WPT smokers (OR: 2.90; 95% CI: 1.05–7.97, p = 0.039).
35	**Okamoto, 2023** ^104^	Japan	Smoking Cross-Sectional Study	Ebina Medical Center, Ebina / N = 965 Jan. 2015–Jun. 2015	**RE**	Compared to never smokers, former and current smokers showed increased risk of RE (OR: 1.5; 95% CI: 0.9–2.4, p = 0.08) and (OR: 2.4; 95% CI: 1.5–3.9, p = 0.01), respectively.
36	**Okello, 2016** ^105^	Uganda	Smoking Case-Control Study	Mbarara Regional Referral Hospital / N = 209 Jan. 2003–Dec. 2014	**ECa**	In multivariate analysis, smoking was not statistically associated with ESCC. According to univariate analysis, smoking was significantly associated with ESCC (OR: 2.93; 95% CI: 1.43–5.71, p = 0.003). On multivariate analysis OR was 1.38 (95% CI: 0.41–4.67, p = 0.600).
37	**Pan, 2019** ^106^	China	Smoking Cross Sectional Study	Huai’an, Jiangsu Province / N = 11518 Jan. 2011–Dec. 2017	**ECa**	Excessive smoking was associated with an increased risk of esophageal precancerous lesions (EPL) Consuming > 30 cigarettes/day was significantly associated with EPL (OR: 1.75; 95% CI: 1.09–2.80). Having 40 or more pack-years of cumulative amount of smoking was also significantly associated with EPL (OR: 1.40; 95% CI; 1.03–1.89).
38	**Park, Kim, Jung, 2022** ^107^	South Korea	Smoking Cohort Study	Korea National Health Insurance Database / N = 43380 2002–2013	**PUD**	Compared to the never-never group, all other groups had significantly adjusted HRs for gastroduodenal ulcer. HR for current-current smokers was 1.379 (95% CI: 1.256–1.513). Heavy smokers had the highest risk, followed by moderate and light smokers.
39	**Park, Kim, Oh, 2022** ^108^	South Korea	Smoking Cohort Study	Korea National Health Insurance Database / N = 97700 2003–2014	**GCa**	Compared to the never-never group, current smokers had higher HRs for gastric Ca. HR for current-current smokers was 1.589 (95% CI: 1.355–1.864). Risk for gastric cancer was highest in heavy smokers, followed by moderate smokers.
40	**Quan, 2015** ^109^	Canada	PM_2.5_, PM_10_ Case-Crossover Study	Calgary (Discovery) and Edmonton (Replication) / N = 1374 and 1159 2004–2010	**PUD**	When air pollution exposures were assessed as 3-, 5-, and 7- day averages, pollutants were inversely associated with UGIB in the discovery cohort. 5-day averages of PM_2.5_ and PM_10_ had ORs of 0.75 (95% CI: 0.61–0.90) and 0.87 (95% CI: 0.75–1.00), respectively.
41	**Rafiq, 2020** ^110^	India	Smoking / Second-Hand Smoking Case-Control Study	Kashmir / N = 2367 Sept. 2008–Jan. 2012	**ECa**	Among never-tobacco users, the association between ever exposure to SHS and ECa risk were (OR: 1.32; 95% CI: 0.43–4.02) Non-smokers exposed to SHS had OR of 1.25 (95% CI: 0.66–2.38), whereas active smokers not exposed to SHS had OR of 1.49 (95% CI: 1.08–2.04).
42	**Ramos, 2018** ^111^	Brazil	Smoking Case-Control Study	Sao Paolo / N = 739 2001–2007	**GCa**	Former and current smokers had ORs of 2.25 (95% CI: 1.53−3.31) and 2.67 (95% CI: 1.72−4.13), respectively. Smoking habit was associated with increased risk in all quartiles of consumption analyzed.
43	**Rao, 2022** ^112^	China	PM_2.5_ Cross-Sectional Study	Fujian Province / N = 5479 Jan. 2016–Dec. 2016	**ECa**	Spatial distribution of hospitalization rate of ECa in 2016 was not consistent with that of concentration of PM_2.5_ in same year. Concentration of PM_2.5_ in 2003 and 2004 had strongest correlation with hospitalization rate of ECa in 2016, with Pearson correlation coefficient r value of −0.365.
44	**Sadafi, 2024** ^113^	Iran	Smoking Cross-Sectional Study	Ravansar / N = 9631 2014–2023	**GERD**	The odds of GERD among current smokers was 23% higher than nonsmokers (OR: 1.23; 95% CI: 1.02–1.55)
45	**Schmidt, 2020** ^114^	Germany	Smoking Case-Control Study	Southern Germany and Augsburg / N = 587 and 1976 2013–2017	**BE**	BE cases were statistically significantly more likely to smoke (32.3% vs 46.1% nonsmokers). Male patients with BE were significantly more likely to smoke (28.2% vs 38.3% nonsmokers) 67.7% of BE cases were ever-smokers.
46	**Seo, 2020** ^115^	South Korea	PM_2.5_, PM_10_ Cross-Sectional Study	Korea National Health Insurance Database / N = 200000 2002–2017	**GERD**	The final model of the study significantly predicted GERD-related medical utilization. In particular, PM_2.5_ and CO were identified as risk factors for GERD.
47	**Sewram, 2016** ^116^	South Africa	Smoking Case-Control Study	3 major public referral hospitals, East Cape Province / N = 1858 Nov. 2001–Feb. 2003	**ECa**	For males, ever smokers had 4-fold increased odds compared to never smokers (OR: 4.11; 95% CI: 2.55–6.65) For females, ever smokers had 3.5-fold increase odd compared to non-smokers (OR: 3.45; 95% CI: 2.47–4.82).
48	**Simba, 2023** ^117^	Kenya, Tanzania, Malawi	Smoking Case-Control Study	Eldoret, Kenya; Moshi, Tanzania; Blantyre, Malawi / N = 623, 1131, 870 Aug. 2013–May 2020	**ECa**	Ever-tobacco use was associated with increased ESCC risk in all countries: Tanzania (OR: 3.09; 95% CI: 1.83–5.23), Malawi (OR: 2.45; 95% CI: 1.80–3.33), and lesser in Kenya (OR: 1.37; 95% CI: 0.94–2.00). Combined OR: 2.15 (95% CI: 1.72–2.68) ESCC risk increased in with tobacco intensity and smoking duration. In all three countries, smoking tobacco showed increased risk of ESCC (OR: 2.28; 95% CI: 1.80–2.89).
49	**Song, 2024** ^118^	South Korea	Smoking Cross-Sectional Study	Seoul National University Bundang Hospital / N = 14598 May 2003–Feb. 2020	**GCa**	In the univariate analysis smoking was significantly associated with single GCa and SGMC in all patients (OR: 0.971; 95% CI: 0.694–1.359) and in EGCa and AGCa patients (OR: 1.200; 95% CI: 0.899–1.602 and OR: 0.468; 95% CI: 0.231–0.949, respectively) Multivariate analysis, smoking was significantly associated with single GCa and SGMC in AGCa patients.
50	**Spreafico, 2017** ^119^	US / Canada	Smoking Cohort Study	Boston, MA and Toronto, Ontario / N = 564 (235; 329) 1999–2004 & 2006–2011	**ECa**	Smoking conferred worse overall survival in the combined Boston-Toronto Cohort with aHR of 1.22 (95% CI: 1.15–1.43)for each 20 pack-year increase.
51	**Sun, 2023** ^120^	China	Smoking / PM_2.5_ Cohort Study	China Kadoorie Biobank / N = 510125 2005–2017	**ECa**	A linear concentration-response relationship between long-term PM_2.5_ exposure and ECa. Each 10-μg/m^3^ increase in PM_2.5_, the HR for ECa was 1.16 (95% CI:1.04–1.30) Using lowest group of PM_2.5_ exposure as reference, HRs for other quartile groups, from low to high, were 1.09 (95% CI: 0.86−1.37), 1.28 (95% CI: 0.98–1.66), and 1.32 (95% CI: 1.01–1.72). Subgroup analyses showed ever smoking had an HR of 1.18 (95% CI: 1.04−1.35).
52	**Thrift, 2022** ^121^	US	Smoking Case-Control Study	Houston, TX / N = 1962	**GCa (IM)**	Compared to never smokers, current smokers had 2-fold increased risk for gastric intestinal metaplasia (OR: 2.05; 95% CI: 1.47–2.85). Among ever smokers, increasing duration and total dose were significantly associated with increased risk (p = 0.004 and 0.01, respectively).
53	**Tsai, 2019** ^122^	Taiwan	PM_2.5_, PM_10_ Case-Crossover Study	Taipei / N = 23205 2009–2013	**PUD**	Increases in both PM_2.5_ (OR: 1.14; 95% CI: 1.09–1.18) and PM_10_ (OR: 1.05; 95% CI: 1.01–1.08) were significantly associated with increased risk of hospital admissions on warm days. On cool days, only increases in PM_10_ were found to be significantly associated with increased risk of hospital admission (OR: 1.04; 95% CI: 1.02–1.07).
54	**Wang, Leena, 2016** ^123^	India	Smoking Cross-Sectional Study	Trivandrum District / N = 1072 2010–2011	**GERD**	No association between cigarette smoking and risk of GERD. For the association of ever-smokers and risk of GERD, a mutually-adjusted analysis yielded OR of 0.7 (95% CI: 0.4–1.2).
55	**Wang, Katki, 2021** ^124^	US	Smoking Cohort Study	NIH-AARP Cohort / N = 490605 1995–2011	**ECa/GCa**	For esophageal cancers, current smoking yielded HRs of 5.75 (95% CI: 3.90–8.49) for ESCC and 3.16 (95% CI: 2.54–3.92) for EADC. For gastric cancers, current smoking yielded HRs of 3.16 (95% CI: 2.42–4.13) for GADC and 1.61 (95% CI: 1.27–2.05) for GNCA.
56	**Wei, 2021** ^125^	China	Smoking Case-Control Study	Feicheng, Shandong / N = 464 Jul. 2013–Apr. 2014	**ECa**	Ever smoking was associated with 3.11-fold increase in ESCC risk (OR: 3.11; 95% CI: 1.63–6.05) For each cigarette-years increase in smoking index, ESCC risk increased by 56% (OR: 1.56; 95% CI: 1.18–2.13).
57	**Wong, 2016** ^126^	China	Smoking / PM_2.5_ Cross-Sectional Study	Hong Kong / N = 66820 Jul. 1998–Dec. 2001	**PUD**	Adjusted HR for PUD hospitalization per 10 μg/m^3^ of PM_2.5_ was 1.18 (95% CI: 1.02–1.36). Associations with PM_2.5_ were significant for gastric ulcers (HR: 1.29; 95% CI: 1.09–1.53) but not for duodenal ulcers (HR: 0.98; 95% CI: 0.78–1.22) For other variables, current smokers were to have significantly increased risk for hospitalization of PUD (HR: 1.59; 95% CI: 1.37–1.84).
58	**Wu, 2021** ^127^	China	PM_2.5_ / PM_10_ Ecological Study	Yinzhou District, Ningbo City, Zhejiang Province / N = 204257 Jan. 2017–Dec. 2019	**PUD**	Cumulative risk ratios for PM_2.5_ and PM_10_ showed nearly linear adverse effect and gently grew to maximums of 2.40 (95% CI: 1.36–4.24) and 1.65 (95% CI: 0.98–2.76), respectively. Significant associations for both men and women were only observed for PM_2.5_.
59	**Yang, Lin, 2020** ^128^	China	Smoking Case-Control Study	Fujian Province / N = 423 Jan. 2010–Dec. 2016	**ECa**	Tobacco smoking was related to ESCC risk, but no significant difference in magnitude of its association with respect to macroscopic type of cancer. Tobacco smoking showed increased risk for ulcerative type ESCC (OR: 2.24; 95% CI: 1.20–4.19) and medullary type ESCC (OR: 2.56; 95% CI: 1.29–5.06).
60	**Yang, Chen, 2017** ^129^	China	Smoking Case-Control Study	Taixing / N = 3314 Oct. 2010–Sept. 2013	**ECa**	In a fully adjusted analysis, current smokers had OR of 1.12 (0.88–1.44) but not significant. Male heavy smokers (i.e., smoked more than 20 cigarettes/day or 40 pack- years or started smoking early) showed a moderately increased risk for ESCC.
61	**Yates, 2014** ^130^	UK	Smoking Cohort Study	EPIC-Norfolk Cohort N = 24068 1993–1997	**BE/ECa**	Hazard ratios for current and former smokers for BE were 1.57 (95% CI: 0.83–2.96) and 1.38 (95% CI: 0.88–2.16), respectively. Hazard ratios for current and formers smokers for EAC were 1.82 (95% CI: 0.81–4.09) and 1.27 (95% CI: 0.71–2.27), respectively. Current and former smoking were not significantly associated with BE and EAC.
62	**Yu, 2021** ^131^	China	PM_2.5_ Cross-Sectional Study	Zhejiang Province / N = 647,092 Jan. 2014–Dec. 2018	**PUD**	A potential dose-response relationship was observed between quartile concentrations of PM_2.5_ 1 month before gastroscopy and detection of PUD. Subjects in the highest quartile of PM_2.5_ exposure displayed significantly higher risk (OR: 1.178; 95% CI: 1.118–1.242). The overall estimated OR for the detection of PUDs associated with a 10 μg/m^3^ increase in PM_2.5_ was 1.050 (95% CI: 1.038–1.063)
63	**Zacharakis, 2023** ^132^	Saudi Arabia	Smoking Cohort Study	Al-Kharj, Riyadh / N = 1080 Jan. 2017–May 2023	**GCa**	Current and former smoking yielded ORs of 4.00 (95% CI: 2.05–7.81) and 0.79 (95%. CI: 0.28–2.24), respectively. Only current smoking was a significant risk factor for GCa (P = 0.002)
64	**Zhao, 2017** ^133^	China	Smoking Case-Control Study	4 counties Jiangsu Province / N = 18093 Jan. 2003–Dec. 2010	**ECa/GCa**	Tobacco smoking was associated positively with both esophageal (aOR: 1.68; 95% CI: 1.50–1.87) and stomach cancer (aOR: 1.61; 95% CI: 1.43–1.81). There was a significant does-response relationship between pack-years of smoking and risks of esophageal (P < 0.001) and stomach cancer(P < 0.001).

## Data Availability

All data generated or analyzed during this study are included in this published article and its supplementary information files.

## Abbreviation

AEE: Asymptomatic Erosive Esophagitis
AGCa: Advanced Gastric Cancer
AHR: Airway Hyperreactivity
aOR: Adjusted Odds Ratio
BE: Barrett’s Esophagus
Ca: Cancer
CC: Correlation Coefficient
Cl: Confidence Interval
E: Esophagitis
EAC: Esophageal Adenocarcinoma
ECa: Esophageal Cancer
EE: Eosinophilic Esophagitis
EPL: Esophageal Precancerous Lesions
ESCC: Esophageal Squamous Cell Carcinoma
GAC: Gastric Adenocarcinoma
GERD: Gastroesophageal Reflux Disease
GCa: Gastric Cancer
GNCA: Gastric Non-Cardia Adenocarcinoma
HR: Hazard Ratio
NOS: New-Castle Ottawa Scale
OAD: Obstructive Airway Disease
OR: Odds Ratio
PAH: Polycyclic Aromatic Hydrocarbons
PEO: Population, Exposure, Outcome
PHE: Potentially Harmful Elements
PM: Particulate Matter
PUD: Peptic Ulcer Disease
RE: Reflux Esophagitis
RR: Risk Ratio
SGMCa: Synchronous Multiple Gastric Cancer
SHS: Second-Hand Smoke
UGIB: Upper Gastrointestinal Bleeding
WHO: World Health Organization
WPT: Waterpipe Tobacco
WTC: World Trade Center
